# Supplementary figures and images for: Towards standardization of measuring anxiety and depression: Differential item functioning for language and Dutch reference values of PROMIS item banks
Source: PLoS One. 2022 Aug 23;17(8):e0273287. doi: 10.1371/journal.pone.0273287 (PMC9398458; doi:10.1371/journal.pone.0273287)

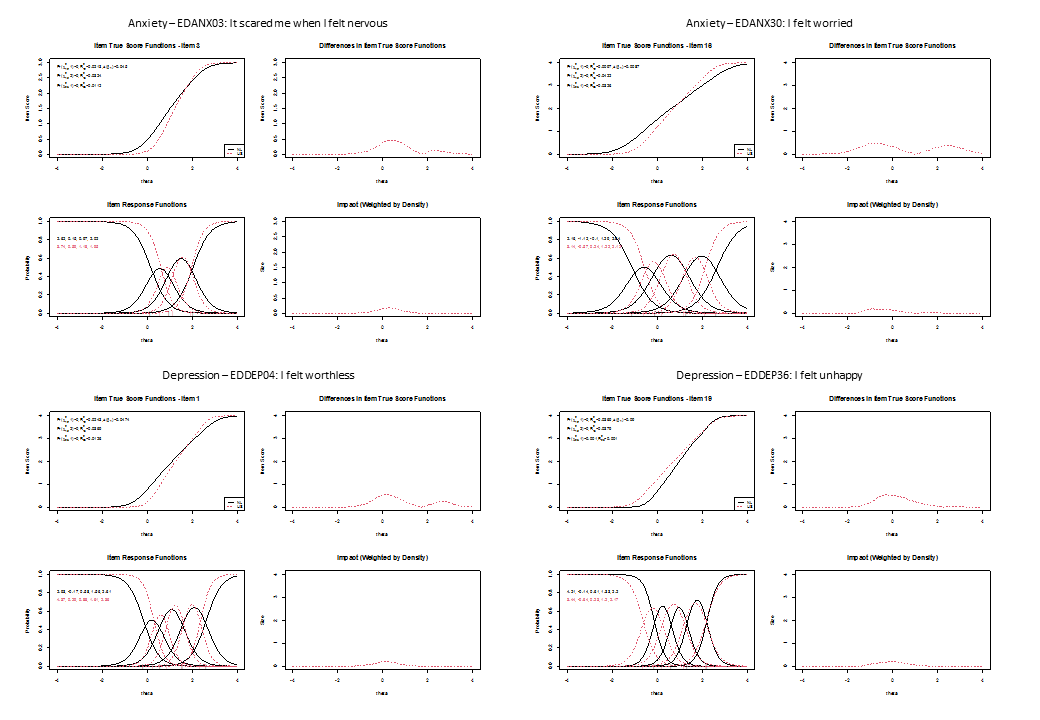

Supplement: S1 Fig — (TIF) [file pone.0273287.s001.tif]

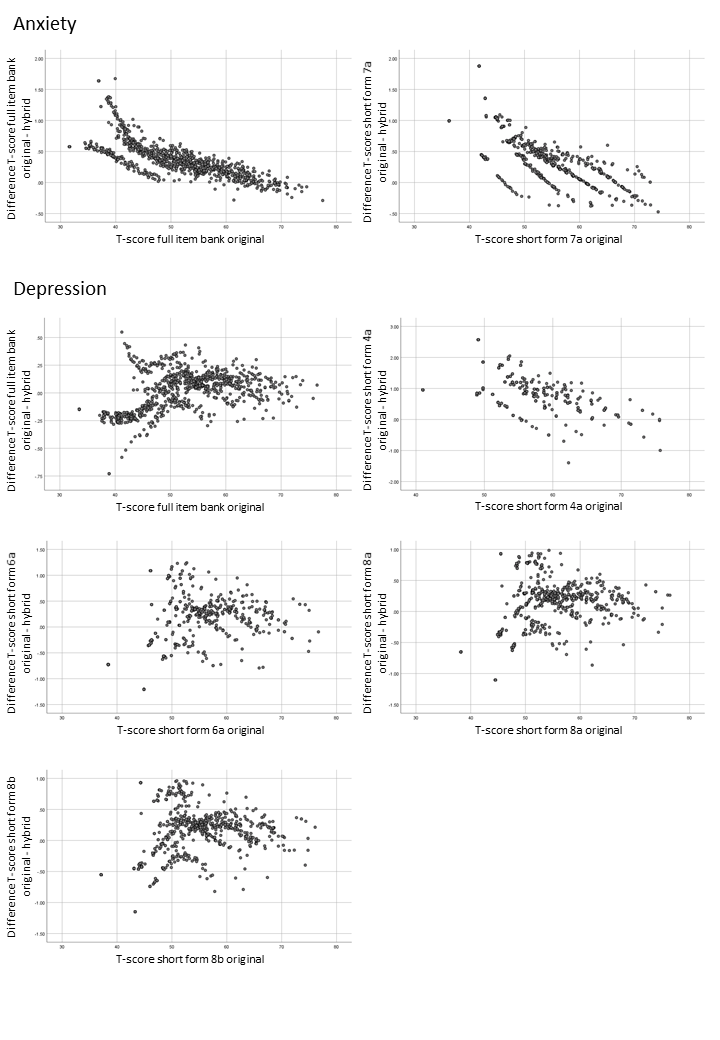

Supplement: S2 Fig — (TIF) [file pone.0273287.s002.tif]
