# Supplementary material for: Towards standardization of measuring anxiety and depression: Differential item functioning for language and Dutch reference values of PROMIS item banks
Source: PLoS One. 2022 Aug 23;17(8):e0273287. doi: 10.1371/journal.pone.0273287 (PMC9398458; doi:10.1371/journal.pone.0273287)
Supplement: Attachment — Submitted filename: Response to Reviewers.docx [file pone.0273287.s006.docx]

PLOS ONE
Associate editor: Jianhong Zhou

22 April 2022

Manuscript ID: PONE-D-21-18689R1
Title: Towards standardization of measuring anxiety and depression: Differential item functioning for language and Dutch reference values of PROMIS item banks

Dear dr. Zhou,

First of all, we would like to thank you for the opportunity to submit a second revised version of our manuscript. We thank the reviewers again for their careful examination of the manuscript and useful suggestions to improve the quality of the paper. Below is our point-by-point response to each of the reviewers’ comments. Our manuscript contains tracked changes to highlight all changes that have been made to the manuscript. Additionally, we have included an unmarked version of the manuscript without tracked changes. Please note that the page numbers in the authors’ reply refer to the revised manuscript with tracked changes.

**Journal Requirements:**

**Please review your reference list to ensure that it is complete and correct. If you have cited papers that have been retracted, please include the rationale for doing so in the manuscript text, or remove these references and replace them with relevant current references. Any changes to the reference list should be mentioned in the rebuttal letter that accompanies your revised manuscript. If you need to cite a retracted article, indicate the article’s retracted status in the References list and also include a citation and full reference for the retraction notice.**

We have reviewed our reference list and updated the references that were previously indicated as ‘submitted’ but at this time have been published. We could not identify any papers that have been retracted in our reference list.

**Review Comments to the Author**

**Reviewer #1:
Thank you for addressing all of my comments. The manuscript is a great contribution to the measurement science literature as well as to researchers and clinicians implementing PROMIS.**

We are pleased to read that we have addressed the reviewer’s comments adequately and for the reviewer’s acknowledgement of the value of our manuscript.

**Reviewer #2: I really appreciate the author’s careful consideration of the reviewers’ comments and clear responses to them. I only have a few additional minor suggestions for further clarification (the indication of page and line numbers refer to the version with track changes):**

We thank the reviewer for these compliments and will respond to the additional suggestions below.

**- Page 5, line 107; and Page9, line 194: The indication that the reference values provided refer to the Dutch general population has been deleted. However, I think it is important to indicate which is the reference population for which the reference values are provided, as reference values (or sub-norms) can be provided for any relevant population (see norms and sub-norms chapter in PROMIS Score and Interpret section of the PROMIS web page : https://www.healthmeasures.net/score-and-interpret/interpret-scores/promis/reference-populations). Therefore, I would keep the indication that reference values obtained are Dutch general population-based reference values for the overall population and by age and gender.**

We thank the reviewer for this remark, and agree that the reference values are established for the Dutch general population and that this notion is important. The reason we changed this is because these reference values, although established for the Dutch general population, are often used in clinical care, to compare scores from clinical populations with. However, we realize that changing these sentences might have led to confusion, and therefore have undone these changes (p. 5, line 104; p. 8, line 182).

**- Page 7, line 143: “expressed as T-scores, with a mean of 50 and a standard deviation of 10 for the US general population [16]”: Please check this reference, as I think it does not refer to scoring interpretation of PROMIS. Should this be reference 17 instead?**

The reviewer is correct, this should be reference 17. We have changed this in the manuscript (p. 7, line 140).

**- Pages 10 (line 216) and 11 (line 220), reference 58: Given this reference is a personal e-mail sent to the author, I don’t think this can be included among the references of the article.**

We have checked the journal requirements and the reviewer is correct. As such, we have deleted this reference from the manuscript (p. 10, lines 204 and 208).

**- Page 12, line 248: “The Stocking-Lord constants were Α=0.7604 and Β=-0.1070 for anxiety and Α=0.7786 and Β=0.0339 for depression”: Following my suggestions the information regarding stocking and lord transformation has been substantially reduced, which I think is Good. Now, I don’t think it is relevant to include the SL constants A and B for anxiety and depression. Otherwise, if included, additional indication should be provided to indicate what A and B mean (perhaps keep the formulas).**

The reviewer makes a valid remark. We have removed the SL constants from the manuscript (p. 11, lines 236-237).

We hope we adequately addressed all points and comments made by the reviewers. Thank you for reconsidering our manuscript. We look forward to receiving your response in due course.

Yours sincerely,

The authors
